# A National Survey Including Data from 5986 Dry Eyes Treated with a Novel Tear Substitute Containing Ribohyal

**DOI:** 10.3390/jcm15145367

**Published:** 2026-07-09

**Authors:** Filippo Lixi, Mihaela-Madalina Timofte-Zorila, Mara-Ioana Tomi, Lorenzo Rapisarda, Marco Messina, Lacrinova Experience Working Group

**Affiliations:** 1 Eye Clinic, Department of Surgical Sciences, University of Cagliari, 09124 Cagliari, Italy; f.lixi0106@gmail.com; 2 Department of Ophthalmology, Grigore T. Popa University of Medicine and Pharmacy Iasi, 700115 Iasi, Romania; madalinatim@yahoo.com; 3 Department of Ophthalmology, Clinical Institute of Ophthalmological Emergencies “Prof. Dr. Mircea Olteanu”, 010464 Bucharest, Romania; mara.tomi@stud.umfcd.ro; 4 Department of Medicine and Surgery, University of Enna “Kore”, Piazza dell’Università, 94100 Enna, Italy; rapisarda.oculisti@gmail.com; 5 Department of Biomedical and Surgical Sciences, Section of Ophthalmology, University of Perugia, S. Maria della Misericordia Hospital, 61029 Perugia, Italy

**Keywords:** Lacrinova, dry eye disease, hyaluronic acid, riboflavin, Ribohyal, artificial tears, real-world study, survey

## Abstract

**Background:** Dry Eye Disease (DED) is a multifactorial condition with an impact on quality of life and an often suboptimal response to conventional lubricants. This study aimed at evaluating the real-world efficacy, tolerability, and patient adherence of a therapy with a novel ophthalmic solution containing Ribohyal, a riboflavin–hyaluronic acid complex, used in patients with DED. **Methods:** This national survey included data from adult patients with clinically diagnosed DED treated with Lacrinova eye drops (4 times/day for 3 months) whose data were collected from 82 Italian ophthalmologists. Patients were assessed before (T0) and after treatment (T1). Outcomes included patient-reported symptoms for 9 domains (0–10 numerical rating scale), objective clinical parameters (best-corrected visual acuity [BCVA], break-up time [BUT], and corneal fluorescein staining [CFS]), and treatment acceptability. **Results:** Data from 2993 patients (5986 eyes) were analyzed. Improvements were observed across all 9 symptom domains, with reductions ranging from 53% to 60% (all *p* < 0.001). BCVA and BUT significantly improved (from 0.08 ± 0.22 at T0 to 0.06 ± 0.19 logMAR at T1 and from 9.20 ± 4.49 to 11.85 ± 4.66 s at T1, respectively, both *p* < 0.001). The prevalence of CFS decreased from 42.0% to 23.4% (*p* < 0.001). Treatment acceptability was high, with mean scores of 8.88± 1.49 for tolerability, 8.55 ± 1.63 for adherence, and 8.62 ± 1.55 for perceived efficacy. No relevant adverse events were reported. **Conclusions:** Lacrinova eye drops demonstrated significant improvements in both symptoms and clinical signs of DED, with excellent tolerability and adherence. These findings support its use as an effective therapeutic option in routine clinical practice, although controlled studies are warranted to confirm the efficacy.

## 1. Introduction

Dry Eye Disease (DED) is one of the most prevalent and complex conditions in modern ophthalmology, with a substantial impact on patients’ quality of life [1,2]. Prevalence estimates vary widely, ranging from 5% to 50% in the general population, and may reach up to 75% among individuals over 40 years of age [3]. According to the TFOS DEWS III definition, DED is “a multifactorial, symptomatic disease characterized by a loss of homeostasis of the tear film and/or ocular surface, in which tear film instability and hyperosmolarity, ocular surface inflammation and damage, and neurosensory abnormalities are etiological factors” [1].

Commonly reported symptoms are dryness, burning, a foreign body sensation and photophobia [1,2]. Beyond its clinical manifestations, DED represents a growing public health concern, particularly in industrialized countries where lifestyle and environmental factors contribute significantly to disease burden. Indeed, increased screen exposure, reduced blink rate, air conditioning, and pollution are now recognized as major contributors to tear film instability and ocular surface stress. Moreover, the progressive aging of the population further amplifies the prevalence and clinical relevance of DED, making it one of the leading causes of ophthalmology consultations worldwide.

DED diagnosis relies on the presence of subjective symptoms combined with objective signs including reduced tear-film break-up time (BUT), corneal fluorescein staining (CFS), and elevated tear osmolarity [1,4].

The cornerstone of DED management is the use of lubricating eye drops. Available products differ in polymers, lipid components, osmolarity, viscosity, preservatives, and dosing characteristics. However, many forms of DED remain challenging to treat, and a proportion of patients continue to experience persistent symptoms despite conventional lubricant therapy [5,6].

To try to address these unmet clinical needs, an innovative ophthalmic solution named Lacrinova (Diadema Farmaceutici, Pisa, Italy) has been developed. It is based on Ribohyal, a novel compound obtained through the conjugation of vitamin B2 (riboflavin) with hyaluronic acid (HA) 0.1%. Ribohyal preserves and enhances the viscoelastic, hydrating, and mechanical-protective properties of HA, while incorporating the antioxidant, photoprotective, and regenerative effects of riboflavin [7]. Upon instillation, the liquid formulation interacts with the ocular surface and forms a thin gel layer, thereby prolonging residence time and providing sustained, intensive hydration without inducing transient visual blurring [7].

The present report describes the results of an Italian survey called “Lacrinova Experience”, an initiative involving 82 Italian ophthalmologists aimed at evaluating the clinical efficacy, tolerability, and patient adherence of this tear substitute in a large cohort of DED patients.

## 2. Materials and Methods

### 2.1. Patients and Data Collection Instrument

In this real-world national survey, data collected from 82 ophthalmologists were analyzed. All enrolled patients received Lacrinova eye drops, and no placebo, vehicle, or active-comparator group was included. Eligible participants were patients aged 18 years or older with a clinical diagnosis of DED based on subjective symptoms and objective signs of disease (BUT < 7 s and/or positive CFS). Exclusion criteria included presence of uncontrolled systemic or ocular conditions and recent ocular surgery (within 1 month).

Patient assessments were conducted at 2 predefined time points: at baseline (T0), and after 90 days of therapy with Lacrinova eye drops (4 instillations per day) (T1). Relevant characteristics recorded for all participants included: age, sex, prior use of artificial tears, history of autoimmune disease, contact lens wear, symptoms and signs of DED.

At both visits, each participating ophthalmologist completed a standardized electronic questionnaire for every enrolled patient. The questionnaire comprised 4 main domains. Domain A (collected at T0 only) captured patient demographics and clinical history, including age group (<31, 31–40, 41–50, 51–60, 61–70, or >70 years), sex, history of auto-immune diseases, habitual contact lens wear, and current or prior chronic use of artificial tears. Domain B assessed patient-reported symptoms using a Numerical Rating Scale (NRS) from 0 (no symptom) to 10 (worst possible), covering nine functional domains: light sensitivity (photophobia), foreign body/gritty sensation, transient visual blurring, difficulty driving at night, discomfort during screen use (computer or mobile phone), dis-comfort while watching TV, discomfort in windy conditions, discomfort in low-humidity environments, and discomfort in air-conditioned environments. Domain C recorded objective clinical parameters including logMAR best-corrected visual acuity (BCVA), BUT, and CFS for each eye. Domain D (collected at T1 only) evaluated patient-reported treatment acceptability across three items, tolerability, compliance with the prescribed regimen, and perceived efficacy, each rated on an NRS from 0 (worst) to 10 (best). The full questionnaire structure is summarized in Table 1.

### 2.2. Statistical Analysis

Statistical analysis was conducted using SPSS for Macintosh software (version 30.0.0.0; IBM Corp., Armonk, NY, USA). Means ± standard deviations (SDs) were calculated for numerical continuous variables, while frequencies and percentages were presented for categorical data. The Shapiro–Wilk test was used to assess data normality.

Within-patient changes in ordinal symptom scores were assessed using the Wilcoxon signed-rank test. Subgroup comparisons on treatment acceptability were performed using non-parametric tests, Mann–Whitney U test or Kruskal–Wallis test, as appropriate. Continuous parameters were analyzed using linear mixed-effects models (LMMs), with visit (time point) and eye (right/left) included as fixed effects and patient as a random effect, assuming a compound symmetry covariance structure to account for within-subject correlation between fellow eyes.

Binary outcomes were analyzed using generalized estimating equations (GEEs) with a binary logistic model, including visit and eye as factors and specifying an exchangeable correlation structure to account for inter-eye correlation within patients. Results are expressed as Odds Ratios (ORs) with 95% Wald Confidence Intervals. A *p* value < 0.05 was considered statistically significant.

## 3. Results

### 3.1. Demographic Data

Overall, 5986 eyes of 2993 patients (1187 males, 1806 females) were included in the study analysis. The majority of patients belonged to the older age groups, with those aged > 70 years representing the largest subgroup (29.7%), followed by patients aged 61–70 years (21.5%). Younger patients (≤30 years) constituted the smallest proportion of the cohort (7.9%). A consistent female predominance was observed across all age groups, with women accounting for approximately 60% of patients in each stratum. The age distribution is detailed in Table 2.

**Figure 1:**
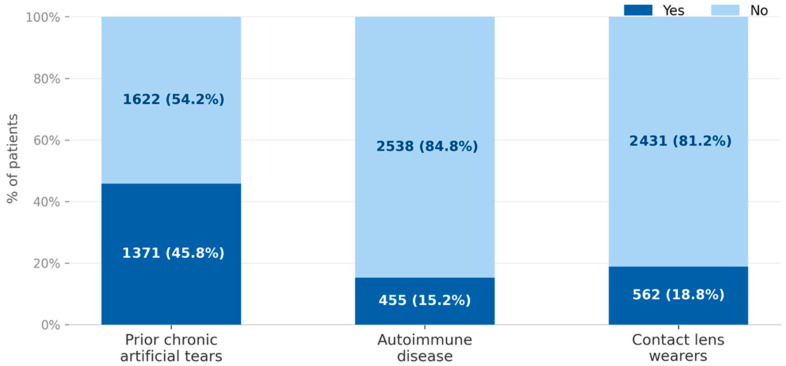
Prevalence of key clinical history variables at T0 (n = 2993). Bars show absolute counts and percentages.

The relevant clinical history is summarized in Figure 1.

### 3.2. Subjective Symptoms

All 9 symptom domains showed statistically significant improvement after 90 days of treatment (Table 3).

### 3.3. Clinical Parameters

BCVA was available for all 2993 patients (5986 eyes), whereas BUT and CFS were assessed and analyzed in a predefined subset of 1602 patients (3204 eyes).

BCVA improved significantly from T0 to T1 (LMM: F(1, 8976) = 53.20, *p* < 0.001). The estimated mean BCVA changed from 0.08 ± 0.22 logMAR at T0 to 0.06 ± 0.19 logMAR at T1, corresponding to a mean change of −0.024 logMAR (95% CI: −0.031 to −0.016). No significant differences were observed between right and left eyes (F(1, 8976) = 1.32, *p* = 0.250), and the visit-by-eye interaction was not significant (F(1, 8976) = 1.71, *p* = 0.191), indicating a comparable improvement in both eyes.

At T0, the mean BUT was 9.20 ± 4.49 s, increasing to 11.85 ± 4.66 s at T1. This change was statistically significant (LMM: F(1, 4803) = 1564.67, *p* < 0.001), with an estimated mean increase of +2.595 s (95% CI: 2.409–2.780). No significant differences were found between right and left eyes (F(1, 4803) = 0.33, *p* = 0.563), and no significant visit-by-eye interaction was observed (F(1, 4803) = 0.63, *p* = 0.428), indicating a consistent effect across both eyes.

CFS was evaluated in 3204 eyes with complete data at both time points. At T0, CFS was present in 1346 eyes (42.0%), decreasing to 751 eyes (23.4%) at T1, corresponding to a relative reduction of 44.2%. GEE analysis with a binary logistic model confirmed a significant reduction in the odds of CFS over time (Wald χ^2^(1) = 295.49, *p* < 0.001). The odds of CFS were significantly higher at T0 compared to T1 (OR = 2.42; 95% CI: 2.18–2.68). No significant differences were observed between right and left eyes (Wald χ^2^(1) = 0.01, *p* = 0.914), and the visit-by-eye interaction was not significant (Wald χ^2^(1) = 1.91, *p* = 0.167), indicating a consistent effect across both eyes.

### 3.4. Treatment Acceptability

Patient-reported treatment acceptability scores were high across all three domains (0-10 scale): tolerability 8.88 ± 1.49, adherence 8.55 ± 1.63, and perceived efficacy 8.62 ± 1.55. No relevant adverse events related to Lacrinova instillation were recorded in either cohort.

Exploratory subgroup analyses were performed to assess whether patient-reported treatment acceptability differed according to baseline demographic and clinical characteristics (Table 4).

Subgroup analyses revealed a significant difference according to age group for all three domains (all *p* < 0.001): mean scores were highest in patients aged < 31 years and 31–40 years and progressively decreased to the lowest values in patients aged > 70 years. Post hoc pairwise comparisons confirmed that the youngest age groups (<31 and 31–40 years) differed significantly from the two oldest groups (61–70 and >70 years) across all three outcomes (all *p* < 0.05), whereas adjacent age groups (51–60 vs. 61–70 years or 61–70 vs. >70 years) did not differ significantly (all *p* > 0.05).

No significant differences in tolerability, adherence, or perceived efficacy were observed according to sex or history of autoimmune disease. In contrast, patients with prior artificial tear use reported higher tolerability (8.99 ± 1.43 vs. 8.79 ± 1.54, *p* < 0.001), adherence (8.66 ± 1.60 vs. 8.46 ± 1.66, *p* < 0.001), and perceived efficacy (8.70 ± 1.52 vs. 8.55 ± 1.58, *p* = 0.010) than patients without prior artificial tears use. Similarly, contact lens wearers reported higher tolerability (9.34 ± 1.08 vs. 8.78 ± 1.56), adherence (9.06 ± 1.34 vs. 8.43 ± 1.68), and perceived efficacy (9.10 ± 1.31 vs. 8.51 ± 1.58) than non-wearers (all *p* < 0.001).

## 4. Discussion

The present survey evaluated the clinical efficacy, tolerability, and patient acceptability after a 3-month therapy with Lacrinova eye drops in a large cohort of patients with DED, providing insights from routine clinical practice conducted across multiple Italian ophthalmology centers. The results demonstrated consistent and clinically meaningful improvements across all outcome domains including subjective symptoms, objective tear film parameters, and ocular surface integrity after 90 days of treatment.

Viewed in the past as mere lubricants designed to transiently supplement tear volume, artificial tears have undergone a substantial paradigm shift over the past decade. Guided by the TFOS DEWS II and III frameworks, next-generation formulations are increasingly conceived as bioactive agents targeting multiple pathophysiological pathways, including oxidative stress, epithelial barrier dysfunction, and neurogenic inflammation [1,8]. Within this landscape, the rationale for Ribohyal-based therapy extends beyond conventional lubrication. Specifically, HA, a naturally occurring glycosaminoglycan present in the ocular tissues, exerts its therapeutic effects by restoring the viscoelastic properties of the tear film, prolonging ocular surface residence time through mucoadhesion, and supporting epithelial integrity by interacting with cell surface receptors involved in wound healing [9,10,11]. Riboflavin, on the other hand, contributes antioxidant and photoprotective activity under conditions of environmental stress, UV exposure, or chronic inflammation [7]. The conjugation of these two molecules into the Ribohyal complex is designed to address both the mechanical and biological dimensions of ocular surface homeostasis [7,9,10,11].

The improvement observed across all patient-reported symptom domains is noteworthy both for its magnitude and its consistency. Reductions ranging from 53% to 60% across diverse functional domains including visually demanding tasks such as screen use and night driving, as well as environmentally triggered discomfort in windy, dry, or air-conditioned settings, suggest a broadly effective mechanism of action rather than one limited to specific symptoms. These findings are consistent with previous reports showing that more than 50% of patients treated with 0.1% HA experienced symptomatic improvement, with foreign body sensation and dryness demonstrating the greatest benefit [12]. Interestingly, HA formulations across a range of concentrations, from 0.1% to 0.4%, have been shown to be effective in improving DED symptoms [9,10,11].

The parallel improvement found in objective findings further support the clinical relevance of the symptomatic response. The observed increase in BUT reflects enhanced tear film stability, consistent with the viscoelastic and mucoadhesive properties of HA [9,10,11]. The marked reduction in CFS prevalence indicates a genuine reparative effect on the ocular surface epithelium. In addition, despite clinically small, the improvement in BCVA is coherent, as enhanced tear film stability reduces wavefront aberrations caused by an irregular ocular surface, thereby translating into measurable functional visual improvement [13,14]. These findings are likely attributable to the combined effects of HA and riboflavin. Indeed, the lubricating and viscoelastic properties of HA promote prolonged retention on the ocular surface, while riboflavin contributes antioxidant protection and supports cellular regeneration [11,15,16].

The acceptability profile of Lacrinova deserves specific attention. In routine clinical practice, non-adherence to prescribed artificial tear regimens represents one of the main obstacles to effective DED management with a reported proportion of only 10.2% of patients who instilled the eyedrops at the frequency prescribed [17]. The high patient-reported scores for tolerability, adherence, and perceived efficacy suggest that the liquid-to-gel formulation, which provides sustained hydration without transient visual blurring upon instillation, may represent a meaningful differentiator in a crowded therapeutic landscape. The absence of relevant adverse events further supports the safety of long-term use in a real-world setting.

Notably, subgroup findings reinforce the notion that Lacrinova is well-tolerated and associated with high adherence across a broad and heterogeneous real-world DED population. Although the absolute differences between age groups were small, a modest but statistically significant age-related gradient was observed, with older patients reporting comparatively lower tolerability, adherence, and perceived efficacy than younger patients. This pattern may reflect age-related differences in symptom perception, expectations of treatment benefit, or practical aspects of eye drop instillation. Moreover, the significantly higher treatment acceptability reported by contact lens wearers may be attributed to their greater familiarity with ocular instillation procedures and generally higher baseline expectations regarding ocular comfort. Similarly, patients with prior artificial tear use reported higher scores across all three acceptability domains compared with treatment-naïve patients. Previous experience with other lubricants may have influenced patients’ expectations and their subjective evaluation of the formulation.

When considered within the broader landscape of DED therapies, Lacrinova occupies a distinct position compared with both conventional lubricants and prescription anti-inflammatory agents. Conventional artificial tears containing carboxymethylcellulose (CMC), polyvinyl alcohol (PVA), or HA primarily aim to improve lubrication, tear-film stability, and ocular surface comfort, although their clinical effects may vary according to composition, concentration, viscosity, and dosing regimen [5,6,9,18]. Conversely, prescription of immunomodulatory agents, such as cyclosporine A 0.05–0.1% and lifitegrast 5%, target the underlying inflammatory cascade and have demonstrated significant improvements in both signs and symptoms of moderate-to-severe DED in randomized controlled trials [5,19,20]. However, these agents are associated with higher costs, the need for a prescription, and in some cases local tolerability issues (burning, stinging) that may limit adherence [5,19,20]. Lacrinova, as an over-the-counter lubricant with added bioactive properties, represents an intermediate option: suitable for mild-to-moderate DED or as adjunctive therapy, with a more favorable tolerability profile and lower barrier to access.

The strengths of this survey include its large sample size, multicenter data collection, and real-world setting, which enhance the generalizability of the findings. However, some limitations should be acknowledged. Firstly, as a prospective observational study without a control arm, causal inference is inherently constrained; placebo effects and regression to the mean cannot be formally excluded. Secondly, the follow-up period was limited to 90 days, and longer-term effects remain to be determined. Thirdly, the lack of stratification by DED severity limits the ability to determine whether treatment effects differ across disease subtypes or severity stages. Fourthly, the real-world recruitment strategy may have introduced selection bias, as patients presenting to specialist centers may not be fully representative of the general DED population. Indeed, the age groups in this cohort were not balanced, with older patients (>60 years) representing the largest subgroup (51.2%) and younger patients (≤30 years) the smallest (7.9%). This skewed distribution may limit the generalizability of findings to younger DED populations.

In conclusion, this large-scale real-world survey demonstrates that Lacrinova eye drops provided clinically relevant and reproducible improvements across a broad spectrum of DED symptoms and objective tear film parameters, with excellent tolerability and adherence in a heterogeneous Italian clinical population. The results support the use of Lacrinova as a valuable therapeutic option for patients with DED in everyday clinical practice. Future studies should aim to investigate the long-term effects of Ribohyal-based drops, as well as their efficacy across different DED subtypes and severity stages. In addition, direct head-to-head comparisons with other artificial tears or anti-inflammatory agents are needed to formally establish the relative efficacy of Ribohyal-based therapy and to better define their positioning within the therapeutic landscape.

## Figures and Tables

**Table 1:** Standardized data collection questionnaire.

#	Item	Response Format
A—Patient demographics and clinical history (collected at T0 only)		
A1	Age	<31/31–40/41–50/51–60/61–70/>70 years
A2	Gender	M/F
A3	History of autoimmune diseases	Yes/No
A4	Habitual contact lens wear	Yes/No
A5	Current or prior chronic use of artificial tears	Yes/No
B—Patient-reported symptoms (NRS 0–10; collected at T0 and T1)		
B1	Eyes that are sensitive to light (photophobia)	NRS 0 (none)–10 (worst)
B2	Eyes that feel gritty/foreign body sensation	NRS 0–10
B3	Blurred vision (transient visual blurring)	NRS 0–10
B4	Problems driving at night	NRS 0–10
B5	Discomfort working with a screen (computer or mobile phone)	NRS 0–10
B6	Discomfort watching TV	NRS 0–10
B7	Discomfort in windy conditions	NRS 0–10
B8	Discomfort in places or areas with low humidity (very dry air)	NRS 0–10
B9	Discomfort in air-conditioned environments	NRS 0–10
C—Objective clinical parameters (collected at T0 and T1)		
C1	Visual acuity—Left Eye	BCVA logMAR
C2	Visual acuity—Right Eye	BCVA logMAR
C3	Fluorescein break-up time (BUT)—Left Eye	Time (seconds)
C4	Fluorescein break-up time (BUT)—Right Eye	Time (seconds)
C5	Corneal fluorescein staining—Left Eye	Yes/No
C6	Corneal fluorescein staining—Right Eye	Yes/No
D—Patient evaluation of treatment (collected at T1 only)		
D1	How do you evaluate the tolerability of the treatment?	NRS 0 (worst)–10 (best)
D2	How do you evaluate your compliance with the prescribed treatment regimen?	NRS 0–10
D3	How do you evaluate the efficacy of the treatment?	NRS 0–10

NRS = Numerical Rating Scale, BCVA = best-corrected visual acuity.

**Table 2:** Age distribution of the population (n = 2993).

Age Group	Total (n)	Total (%)	Male (%)	Female (%)
≤30	238	7.9%	93 (39.1%)	145 (60.9%)
31–40	352	11.8%	134 (38.1%)	218 (61.9%)
41–50	376	12.6%	149 (39.6%)	227 (60.4%)
51–60	495	16.5%	199 (40.2%)	296 (59.8%)
61–70	642	21.5%	252 (39.3%)	390 (60.7%)
>70	890	29.7%	360 (40.4%)	530 (59.6%)

**Table 3:** Changes in symptom scores from T0 to T1 (0–10 scale). ^‡^ = Wilcoxon signed-rank test.

Symptom Domain	T0	T1	Δ%	*p*-Value
Light sensitivity	4.74 ± 2.83	2.24 ± 2.18	−53%	<0.001 ^‡^
Foreign body sensation	5.42 ± 2.74	2.25 ± 2.09	−58%	<0.001 ^‡^
Transient visual blurring	4.35 ± 2.92	1.97 ± 2.09	−55%	<0.001 ^‡^
Night-driving difficulty	4.07 ± 3.03	1.85 ± 2.08	−55%	<0.001 ^‡^
Digital-screen difficulty	4.76 ± 2.90	1.92 ± 2.03	−60%	<0.001 ^‡^
TV-watching discomfort	4.59 ± 2.91	1.86 ± 2.03	−59%	<0.001 ^‡^
Wind-exposure discomfort	5.10 ± 2.79	2.12 ± 2.07	−58%	<0.001 ^‡^
Low-humidity discomfort	4.64 ± 2.87	1.93 ± 2.03	−58%	<0.001 ^‡^
Air-conditioning discomfort	4.97 ± 2.85	1.99 ± 2.06	−60%	<0.001 ^‡^

**Table 4:** Tolerability, adherence, and perceived efficacy by clinical subgroup (0–10 scale). # = Mann–Whitney U; ° = Kruskal–Wallis test.

Subgroup	N (%)	Tolerability Mean ± SD	Adherence Mean ± SD	Perceived Efficacy Mean ± SD
Age				
<31 yr	238 (7.9%)	9.15 ± 1.19	8.75 ± 1.63	8.87 ± 1.47
31–40 yr	352 (11.8%)	9.23 ± 1.38	8.99 ± 1.42	9.03 ± 1.34
41–50 yr	376 (12.6%)	9.09 ± 1.36	8.89 ± 1.48	8.89 ± 1.47
51–60 yr	495 (16.5%)	8.90 ± 1.49	8.55 ± 1.61	8.61 ± 1.56
61–70 yr	642 (21.5%)	8.75 ± 1.54	8.40 ± 1.71	8.51 ± 1.58
>70 yr	890 (29.7%)	8.67 ± 1.59	8.30 ± 1.67	8.37 ± 1.60
*p*-value °		<0.001	<0.001	<0.001
Sex				
Female	1806 (60.3%)	8.88 ± 1.53	8.55 ± 1.63	8.63 ± 1.53
Male	1187 (39.7%)	8.88 ± 1.44	8.55 ± 1.65	8.61 ± 1.58
*p*-value #		0.772	0.718	0.723
Autoimmune disease				
Yes	455 (15.2%)	8.80 ± 1.53	8.44 ± 1.75	8.52 ± 1.64
No	2538 (84.8%)	8.90 ± 1.49	8.57 ± 1.61	8.64 ± 1.54
*p*-value #		0.132	0.294	0.281
Prior artificial tears				
Yes	1371 (45.8%)	8.99 ± 1.43	8.66 ± 1.60	8.70 ± 1.52
No	1622 (54.2%)	8.79 ± 1.54	8.46 ± 1.66	8.55 ± 1.58
*p*-value #		<0.001	<0.001	0.010
Contact lens wear				
Yes	562 (18.8%)	9.34 ± 1.08	9.06 ± 1.34	9.10 ± 1.31
No	2431 (81.2%)	8.78 ± 1.56	8.43 ± 1.68	8.51 ± 1.58
*p*-value #		<0.001	<0.001	<0.001

## Data Availability

The data presented in this study are available on request from the corresponding author.
